# Euler buckling and nonlinear kinking of double-stranded DNA

**DOI:** 10.1093/nar/gkt739

**Published:** 2013-08-16

**Authors:** Alexander P. Fields, Elisabeth A. Meyer, Adam E. Cohen

**Affiliations:** ^1^Biophysics Program, Harvard University, 12 Oxford Street, Cambridge, MA 02138, USA, ^2^Department of Chemistry and Chemical Biology, Harvard University, 12 Oxford Street, Cambridge, MA 02138, USA and ^3^Department of Physics, Harvard University, 12 Oxford Street, Cambridge, MA 02138, USA

## Abstract

The bending stiffness of double-stranded DNA (dsDNA) at high curvatures is fundamental to its biological activity, yet this regime has been difficult to probe experimentally, and literature results have not been consistent. We created a ‘molecular vise’ in which base-pairing interactions generated a compressive force on sub-persistence length segments of dsDNA. Short dsDNA strands (<41 base pairs) resisted this force and remained straight; longer strands became bent, a phenomenon called ‘Euler buckling’. We monitored the buckling transition via Förster Resonance Energy Transfer (FRET) between appended fluorophores. For low-to-moderate concentrations of monovalent salt (up to ∼150 mM), our results are in quantitative agreement with the worm-like chain (WLC) model of DNA elasticity, without the need to invoke any ‘kinked’ states. Greater concentrations of monovalent salts or 1 mM Mg^2+^ induced an apparent softening of the dsDNA, which was best accounted for by a kink in the region of highest curvature. We tested the effects of all single-nucleotide mismatches on the DNA bending. Remarkably, the propensity to kink correlated with the thermodynamic destabilization of the mismatched DNA relative the perfectly complementary strand, suggesting that the kinked state is locally melted. The molecular vise is exquisitely sensitive to the sequence-dependent linear and nonlinear elastic properties of dsDNA.

## INTRODUCTION

DNA bending is an integral part of many biological processes, including genome storage, genetic recombination, mismatch repair and transcriptional regulation (1–3). Quantitative descriptions of these processes require a detailed understanding of the energetics of DNA bends. One would like to know how the bending energy depends on curvature under physiological conditions: at high curvature, does the DNA bend smoothly, or does it kink like a drinking straw?

Furthermore, when viewed up close, DNA is not a monolithic material. Intrinsic curvature (4) and linear (5) and nonlinear bending moduli depend on the underlying sequence, and are additionally affected by interactions with small molecules (e.g. ions and drugs) and by chemical modifications (e.g. mismatches, epigenetic marks and damage). These aspects of DNA mechanics are likely to influence protein binding and DNA packaging, yet are only accessible in measurements where the curvature is localized to a short region of DNA. Here, we present a simple molecular platform for studying these phenomena in a quantitative way. We identify an ionic strength-induced transition between linear elastic bending and nonlinear ‘kinking’, and we study the effect of single-nucleotide mismatches on DNA bending. Our data support a model of linear elastic bending at low curvature, with kinking at high curvature and high ionic strength facilitated by local melting of the duplex.

Double-stranded DNA (dsDNA) bending has been investigated experimentally by transient electric birefringence, solution cyclization, single-molecule stretching, atomic force microscopy, NMR, x-ray crystallography and small-angle x-ray scattering (SAXS) (2,6). The majority of these experiments have borne out the predictions of the worm-like chain (WLC) model, in which bending energy increases quadratically with curvature (7). The WLC is specified by a single parameter known as the persistence length, the characteristic distance over which thermal bending occurs. Polymers shorter than their persistence length behave as elastic rods, and polymers significantly longer behave as random walks. Values for the persistence length of duplex DNA under roughly physiological conditions range from 44–55 nm (6,8), with variation due to salt concentration (9) and sequence (5). We use a consensus value of 46.5 nm, which is consistent with the results of solution cyclization (2) and single-molecule stretching experiments (9).

Despite the success of the WLC model, nearly all experiments on DNA flexibility remain limited to thermally accessible bending energies, leaving the regime of greater curvature comparatively unexplored. The range of bending angles over which the WLC model retains validity is a topic of longstanding and ongoing debate. A recent review emphasized that the conditions favoring putative kinks in DNA are still poorly understood (10). Crick first suggested that the bending energy may be significantly lower than the WLC prediction when the bend angle is large, giving rise to stable kinking of DNA under sufficient imposed curvature (11). Consistent with this hypothesis, 63–66 base-pair (bp) DNA minicircles were found to be digested by single-strand-specific nucleases, suggesting that bending and/or torsional stress induced local structural disruptions (12). A recent study used single-molecule Förster Resonance Energy Transfer (FRET) to measure cyclization of sub-persistence length linear dsDNA with complementary single-stranded overhangs. The observed cyclization rate exceeded the predicted rate from the WLC model (13). On the other hand, SAXS data on DNA as short as 42 bp was consistent with the WLC (14). The differing results may arise because the cyclization data were dominated by rare extreme conformational fluctuations, whereas the SAXS data were dominated by the most prevalent conformations in the ensemble.

## MATERIALS AND METHODS

### Synthesis

All DNA oligonucleotides were purchased from Integrated DNA Technologies Inc. Each hairpin was purchased in three pieces: the two complementary halves of the stem, and the loop. A 5′ terminal phosphate was included on the appropriate strands to enable ligation. Each stem strand included an amine-modified C6 deoxythymidine residue at the end next to the ligation site (these become the two outermost bases of the loop). Strands were separately labeled with Cy3B (GE Healthcare PA63101) or Alexa Fluor 647 (Invitrogen A20006) succinimidyl esters according to manufacturer protocols. We selected Cy3B as the donor dye in place of the more popular Cy3 to avoid variations in quantum yield owing to sequence- and hybridization-dependent photoisomerization (15). Labeled strands were purified from unlabeled strands by reverse-phase HPLC. Two splint strands, each complementary to the entirety of one of the stem strands and the neighboring 10 bases of the loop strand, were separately mixed with their complementary stem strands in ligase buffer. These stem solutions were then combined with each other, the loop strand and T4 DNA ligase (New England Biolabs M0202S). Following reaction overnight at 16°C, double ligation products were purified by denaturing (7 M urea) polyacrylamide gel electrophoresis (PAGE) and extracted using the crush-and-soak method. Sequences are listed in Supplementary Methods.

### Data acquisition

Hairpins were diluted to a final concentration of 1.5 nM and mixed with an excess (circa 100 nM) of the appropriate target strand in PAGE loading buffer containing 10% glycerol (by volume), 89 mM tris/borate (pH 8.3), and 1 µM of a noncomplementary oligonucleotide (to block nonspecific interactions). Samples were annealed by heating to 95°C for at least 5 min, followed by cooling to 25°C at a rate of 1°C/45 s. The native running buffer consisted of 89 mM tris/borate. Native PAGE gels (7.5%) were cast in-house in native running buffer. The samples were loaded and run for 1 h at 150 V. To test the effects of ionic strength on conformation, gels were soaked in a series of buffers containing 89 mM tris/borate and varying concentrations of sodium chloride and/or magnesium chloride. Following each 20-min soak, gels were imaged on a Typhoon Trio (GE Healthcare), with excess buffer included to avoid changes in ionic strength owing to evaporation during the scan. We simultaneously collected two fluorescence images using 532 nm excitation, A_GG_ representing green emission (565–595 nm) and A_GR_ representing red emission (655–685 nm). We then took a third image under 633 nm excitation using the red emission filter, A_RR_. All images were taken at 100 µm pixel spacing using a PMT gain of 750 V. FRET values were extracted from images as described in Supplementary Methods.

### S1 nuclease digestion

Aliquots of the 36-nt loop hairpin were diluted to a concentration of 1 nM with an excess (circa 1 mM) of the appropriate target strand in 10 µl reaction buffer (100 mM NaCl, 3 mM MgCl_2_, 1 mM ZnCl_2_ and 89 mM tris/borate; pH 8.3). We note that Zn^2+^ has been found to facilitate kinking in atomic force microscopy experiments (16), but include it to facilitate S1 nuclease reactivity (17). Samples were annealed by heating to 95°C for 5 min, followed by cooling to 25°C at a rate of 1°C/45 s. Reactions were initiated by addition of 356 units S1 nuclease (Promega M5761) in 10 µl of identical buffer. High S1 concentration was required because the buffer differed from the standard S1 reaction buffer, resulting in a loss of activity. Four 5-µl portions of each reaction mixture were terminated at set time points (5, 15, 45 or 135 min) by dilution into 10 µl denaturing buffer (8 M urea, 80 mM EDTA and 0.002% bromophenol blue) and immediate heat denaturation (20 min at 95°C). Samples were analyzed by denaturing (7 M urea) 9% PAGE imaged on a Typhoon Trio imager as above.

## RESULTS

### Molecular vise for compressing DNA

**Figure 1.**
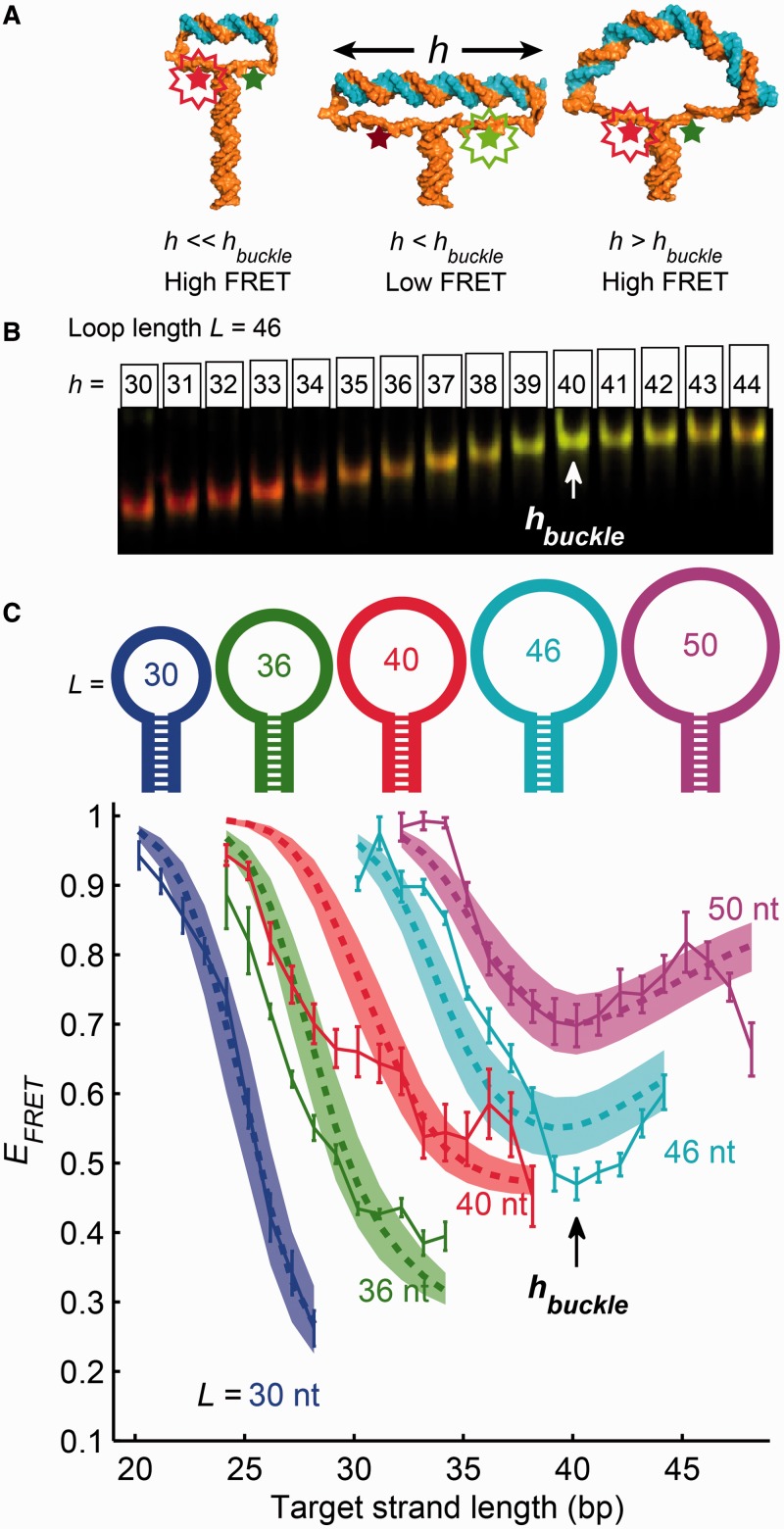
Molecular vises were used to apply compressive forces to short strands of duplex DNA. (**A**) Possible conformations of a molecular vise. The base-pairing force in the hairpin stem (composed of all A-T base pairs) imparted a roughly constant compressive force on the ends of the target strand (top duplex). When the target strand was shorter than the buckling length (left and center images), it withstood the compressive force and remained rigid. The dye separation increased as the target strand grew longer, resulting in a decrease of FRET efficiency. Past the buckling transition (right image), the target strand bent under the compressive force and the FRET efficiency recovered. Molecular cartoons were generated using Nucleic Acid Builder (18) and PyMOL (19). (**B** and **C**) Combined measurements of electrophoretic mobility and FRET. Molecular vises of five loop sizes (30, 36, 40, 46 and 50 nt) were each bound with varying-length target strands, analyzed by native PAGE, and imaged on a commercial scanner (B). FRET efficiency was quantified from gel images and plotted for each loop size as a function of the target strand length (C). The local minimum in FRET efficiency at a target strand length of 40 bp signified the buckling transition and was consistent with the predictions of a statistical mechanical model (shaded areas; see Supplementary Methods for details). The buckling transition also manifested as a change in the dependence of electrophoretic mobility on target length (B; quantified in Supplementary Figure S2). Error bars in (C) are SEM from four independent replicates.

Our ‘molecular vise’ DNA nanostructures (Figure 1) used the free energy of hybridization, rather than thermal fluctuations, to drive bending of a distinct short segment of DNA. The structures consisted of a hairpin with a loop of length 30–50 nt and a stem of length 49 bp. The loop sequences were designed to minimize secondary structure as predicted by M-fold (20). In the stem, the 39 bp adjacent to the loop were all A-T base pairs, to maintain an approximately constant unzipping force. A pair of fluorescent dyes, Cy3B and Alexa Fluor 647, were attached to the nucleotides at the junction of the loop and the stem. The efficiency of FRET between the dyes reported the degree of unzipping of the stem (see ‘Materials and Methods’ section).

The hairpins were divided into aliquots and hybridized with oligonucleotides of variable length, complementary to the apex of the loop. We called the resulting double-stranded segment of the loop the ‘target strand’. All target strands contained entirely G-C nucleotides beyond the central 18 nt, to ensure robust hybridization of the target strand and to avoid fraying at its ends. We varied the loop length, *L*, and the target strand length, *h*, and we recorded the FRET efficiency, 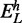, as a function of both parameters.

Figure 1A illustrates plausible conformations of the molecular vise for varying target strand lengths at fixed loop length. Short target strands did not significantly stretch the loop: the stem remained fully zipped and the FRET was high. When the length of the target strand exceeded approximately half the contour length of the loop, the single-stranded regions became taut, the stem began to unzip and the FRET decreased. The tension in the single-stranded regions led to a compressive force on the target strand. Owing to the 100% A-T composition of the top of the stem, the compressive force was nearly constant, independent of the extent of unzipping. Laser tweezers experiments have measured the A-T unzipping force to be 9 pN (21).

When the target strand reached a critical length, it could no longer support the 9 pN compressive load. The target strand then became bent, a phenomenon called Euler buckling. Further increases in the contour length of the target strand led to a *decrease* in its end-to-end separation, and an *increase* in FRET. Thus we expected buckling of the target strand to manifest as a minimum in the plot of FRET efficiency versus length of the target strand. Note that owing to the single-stranded tethers, the ends of the target strand are not subject to torsion about the strand axis. The DNA vise thereby induces pure bending, without twist. This situation differs from experiments on circular DNA [either cyclization (5) or cleavage of minicircles (12)], where the requirement of circular continuity imposes a torsional constraint.

A simple estimate predicts the length of the target strand at the Euler buckling transition. Let us approximate the target strand as a linear elastic rod. For this estimate, we neglect thermal fluctuations because the target strand is much shorter than the persistence length. The Euler buckling force for a straight elastic rod of length *h* is
(1)
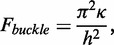

where *κ* is the bending modulus (equal to *l_p_ k_B_ T*, where *l_p_* is the persistence length, *k_B_* is the Boltzmann constant and *T* is the absolute temperature) (22). In a molecular vise, the compressive force is fixed at the 9 pN base-pairing force, but the length of the target strand can vary. We therefore considered the buckling length at constant force,
(2)
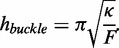

Rods shorter than this length can support the compression; rods longer cannot and instead adopt bent conformations known as elastica (Supplementary Figure S1). For a persistence length of 46.5 nm and a base-pairing force of 9 pN, Equation 2 predicts the buckling length to be 14.5 nm (42.5 bp). This estimate is based purely on continuum mechanics, and neglects thermal fluctuations as well as all molecular details. Below we present a more detailed statistical mechanical model that incorporates these effects.

### Euler buckling of DNA at low salt

We synthesized five molecular vises with loop sizes *L* = 30, 36, 40, 46 and 50 nt, all with the same stem, and mixed aliquots of each with a series of complementary strands of varying length *h*. Samples were analyzed by native PAGE and imaged on a commercial fluorescence scanner (Figure 1B). PAGE was used to segregate the desired construct from molecular aggregates. Hybridization of the target was confirmed by a target length-dependent decrease in mobility relative to the unhybridized hairpin. FRET efficiencies were extracted from gel images using a custom algorithm (Supplementary Methods) and plotted as a function of the length of the target strand (Figure 1C).

In the three smaller loops (30, 36 and 40 nt), the target strand was always shorter than the predicted buckling length. The FRET efficiency decreased monotonically with increasing complement length, consistent with the target strand remaining straight and unzipping the stem. In the larger loops (46 and 50 nt), the FRET efficiency showed a local minimum at a complement length of 40 or 41 bp. The complement length at which this transition occurred was in good agreement with the buckling length predicted by linear elasticity (Equation 2). The transition in FRET also coincided with a transition in electrophoretic mobility: for targets <40 bp, mobility and target length were inversely related, whereas for targets >40 bp, mobility was nearly independent of target length, suggesting a more compact conformation for the assembly (Supplementary Figure S2).

We constructed a coarse-grained statistical mechanical model of the molecular vises to predict the shapes of the FRET curves. The model included three types of energy: the base-pairing energy within the stem and within the target strand (discretized at the single-bp level), the force-extension energy of the unhybridized single-stranded DNA and the bending energy of the double-stranded target strand (calculated via continuum mechanics). The treatment of the target strand in our model is equivalent to the WLC. We calculated the thermal equilibrium ensemble of molecular conformations, and from this, the ensemble-average FRET for each loop and complement combination.

The parameters of the model, including the Förster radius and all mechanical properties, were set to plausible values based on literature sources (Supplementary Table S1). We applied a linear scale factor for each loop length to adjust the range of the FRET curve as the only adjustable parameter in the fits. This simple model lacked numerous effects that may significantly affect FRET efficiency, such as fluctuations of the dye linkers and orientations, long-range electrostatic interactions and all atomic-scale structural details. Details are included in Supplementary Methods.

The predictions of our simple model reproduced the essential features of the experimental data (Figure 1C), including the monotonic loss of FRET with increasing complement length in the three shorter hairpins, and the local minimum in FRET at the buckling transition length of 40 bp (thermal fluctuations shortened the predicted buckling length slightly relative to the purely mechanical estimate). The shaded regions depict the uncertainty in the model owing to uncertainty in literature values of physical parameters. The overall agreement between the predicted and measured FRET curves, despite the simplifications in the model, provides strong support for the WLC picture of DNA bending. Our data thus verify the WLC at curvatures up to 7°/bp (Supplementary Figure S3), significantly beyond the highest curvature probed by bulk cyclization experiments [3.4°/bp (23)] or by recent single-molecule cyclization experiments [5.4°/bp (13)]. Deviations from the WLC, particularly a softening at high curvature, would manifest as a minimum in the FRET efficiency at a shorter target strand length than we observed.

In some regions, the FRET curves deviated from the model. The slight discrepancy between predicted and measured FRET for short targets in the small loops is likely owing to steric clash between the double-stranded target region and the inward-pulling single stranded parts of the loop, an effect not included in the model. The loss of FRET for the longest complement lengths (46–48 bp) in the 50 nt loop was not predicted by our simple model. The concomitant decrease in electrophoretic mobility (Supplementary Figure S2) suggested a structural rather than photophysical basis for the effect; we hypothesize that this deviation may be due to electrostatic repulsion between the ends of the buckled target strand, inhibiting them from nearing each other.

### Salt-induced nonlinear kinks

Under the ionic conditions of our experiments, the double-stranded persistence length is expected to be largely independent of the concentration of sodium or magnesium (6,9). However, the persistence length only reflects the low-curvature bending modulus; the effects of ionic strength on DNA bending at high curvature have not been explored. Recent measurements reporting enhanced flexibility of DNA at short length scales were conducted in unphysiologically high sodium or magnesium, raising the question of whether these results apply under physiological conditions (13).

**Figure 2.**
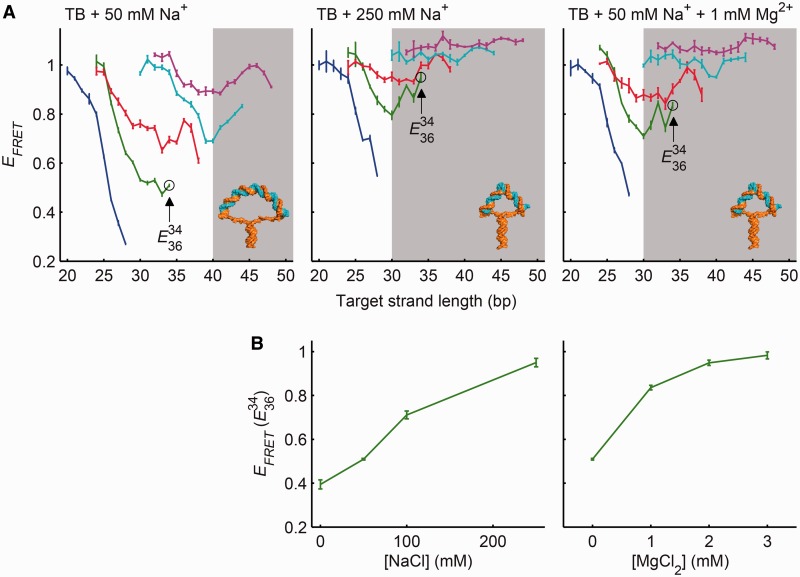
Effect of ionic strength on duplex DNA bending in molecular vises. (**A**) FRET efficiency as a function of target strand length for five loop sizes (colors as in Figure 1C). Addition of 50 mM NaCl did not shift the buckling transition from its original position at 40 bp (left). The presence of 250 mM NaCl (center) or of 50 mM NaCl and 1 mM MgCl_2_ (right) led to a new bending transition at a target strand length of 30 bp and a loss of contrast in the original buckling transition at 40 bp. The transition at 30 bp is inconsistent with Euler buckling and represents a physically distinct bending mode, which we term ‘kinking’. (**B**) The transition of the dominant bending mode from Euler buckling to kinking was quantified by 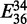 (as indicated in A). In a background of 89 mM tris/borate, the transition occurred between 50 and 100 mM NaCl (left). In 89 mM tris/borate and 50 mM NaCl, the transition occurred on addition of 1 mM MgCl_2_ (right), indicating that Mg^2+^ stabilized the kinked structure significantly more efficiently than did Na^+^. Full FRET curves for all ionic conditions are in Supplementary Figure S2. Error bars are SEM from at least three independent replicates.

We used molecular vises to measure the effect of sodium or magnesium (in a background of 89 mM tris/borate) on the bending rigidity of intact duplex DNA (Figure 2 and Supplementary Figure S4). At 50 mM Na^+^, the buckling transition at 40 bp was preserved, but at 250 mM Na^+^ or 1 mM Mg^2+^, the FRET curve was dramatically different. A bending transition occurred at a target strand length of 30 bp in the loops of length 36 and 40, and the FRET remained high at all target strand lengths for loops of length 46 and 50. At intermediate values of ionic strength, FRET minima were observed at 30 bp in loops of length 36 and 40 and at 40 bp in loops of length 46 and 50 (Supplementary Figure S4), suggesting the existence of two distinct modes of bending. We quantified the transition between the two modes by the FRET efficiency of a complex (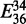), which showed low FRET in low salt and high FRET in high salt (Figure 2B). The transition occurred between 50 and 100 mM NaCl (in 89 mM tris/borate) or at approximately 0.5 mM MgCl_2_ (in 89 mM tris/borate and 50 mM NaCl).

We asked whether the influence of salt could be accounted for by tuning parameters of our WLC-based statistical mechanical model or by varying the unzipping force in the stem. We simulated the effects of increased salt on base-pairing force, single-stranded persistence length and double-stranded persistence length using the known salt-dependence of these parameters (24). The simulations did not match the observed FRET behavior (Supplementary Figure S5A). Indeed, to achieve a buckling length of 30 bp would require a decrease in persistence length of the target to 25 nm, well below all literature values (Supplementary Figure S5B). Thus, at high ionic strength, the dsDNA bent more than predicted by the WLC model.

We thus hypothesized that high ionic strength stabilized a kinked state of DNA, which did not manifest under thermally accessible curvatures. Localized curvature-induced melting has been proposed as a molecular mechanism for nonlinear kinking in dsDNA (25,26). In this ‘meltable WLC’ (MWLC) model, the dsDNA forms a short bubble of two ssDNA strands with a correspondingly shorter persistence length. The MWLC model preserves the bulk persistence length because thermally induced bubbles form at negligible density in unstrained DNA.

The free energy to form a kink has two components:
(3)
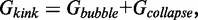

where 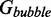 is the free energy cost (positive) to form a sharply bent region of locally melted DNA, and 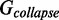 is the free energy gain (negative) by conformational relaxation in the rest of the molecular vise. 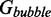 has three contributions: (i) loss of base-pairing energies within the bubble; (ii) a correction for the pinned ends of the bubble [called the ‘cooperativity factor’ (27,28) and ‘loop factor’ (29)]; and (iii) bending of the locally melted region (30). 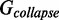 also has three components: (i) relaxation of the curvature in the double-stranded regions of the target; (ii) relaxation of the stretch of the single-stranded tethers; and (iii) zipping of the stem. We applied our statistical mechanical model (Supplementary Methods) to estimate 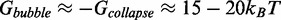 under the conditions where kinking was observed. While the parameters used in the calculation are uncertain, the fortuitous similarity in magnitude of 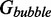 and 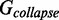 likely accounts for the sensitive salt-dependent equilibrium between buckling and kinking. The dominant effect of salt is to favor the kinked state through an increase in the free energy gains from zipping the stem and relaxing the single-stranded tethers.

The absence of a FRET minimum in loops of length 46 and 50 at high ionic strength is also accounted for by the MWLC model. For these loops, the kinking transition at 30 bp occurs at a shorter target strand length than the minimum target strand length needed to induce a decrease in FRET (∼34 and 35 bp respectively, Figure 1C).

### Flexibility of mismatched DNA

We next applied molecular vises to study the flexibility of DNA containing single base-pairing mismatches. These experiments served two functions. First, they tested the MWLC model, which predicts that the more unstable the mismatch, the less the additional cost to nucleate a bubble, and the greater the propensity to kink. Second, these experiments tested whether mismatch flexibility is correlated with efficiency of mismatch recognition and repair *in vivo* (31,32). This second question connects the underlying biophysics of mismatched DNA to an important process for cell survival and, ultimately, evolution.

Single mismatches are insufficient to alter the equilibrium conformation of bare unstrained dsDNA significantly from the standard B-form (31,32). Solution NMR has found varying levels of increased conformational heterogeneity at mismatch sites (31); however, these experiments only probed thermally accessible conformations, and spectra could not be related directly to curvature of the DNA backbone. Others have inferred mismatch flexibility from sensitivity to T4 endonuclease VII cleavage (33), but this assay may depend on the specifics of the nuclease–DNA interaction rather than on the intrinsic properties of the DNA. Under native conditions, mismatched DNA shows similar electrophoretic mobility to intact DNA, but under slightly denaturing conditions mismatches decrease the electrophoretic mobility, suggesting a mismatch-induced conformational change (34).

**Figure 3.**
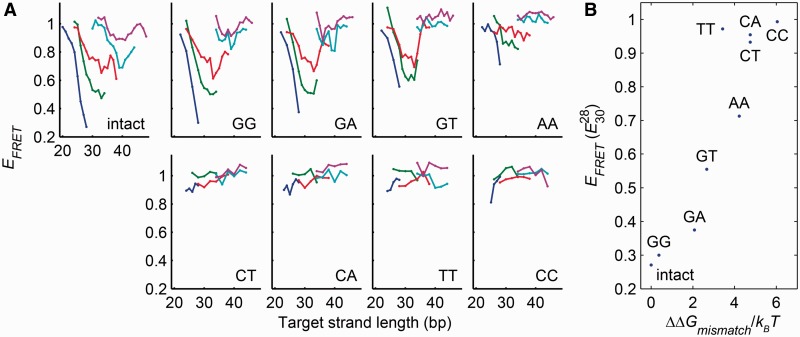
Relative flexibility of base-pairing mismatches. (**A**) FRET efficiency as a function of target strand length for target strands containing each of the eight possible single base-pairing mismatches. Each mismatch was probed in molecular vises with five loop sizes (colors as in Figure 1C). The bending rigidity ranked: {intact, GG, GA} > {GT, AA} > {CT, CA, TT, CC}. (**B**) Flexibility of the mismatches, as quantified by 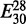, correlated significantly with the free energy penalty of the mismatch (35) (Spearman’s *ρ* = 0.90; *P* = 2.2 × 10^−3^).

To investigate the flexibility of mismatched DNA in the absence of protein binding or denaturants, we introduced each of the eight possible single mismatches at positions located within one nucleotide of the apex of the target strand (see Supplementary Methods for sequences). In all cases, the presence of the target strand shifted the electrophoretic mobility of the vise, confirming that the target hybridized to the loop. We measured the FRET efficiency as a function of target strand length and loop length (Figure 3A), under ionic conditions where perfectly complementary targets showed WLC behavior. We used 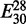 as a qualitative measure of flexibility, with greater FRET indicating greater flexibility.

Four mismatches (T-T, C-T, C-A and C-C) showed little FRET contrast at any loop or complement length, suggesting that under a compression of 9 pN they kinked at a contour length <15 bp. The G-T and A-A mismatches demonstrated intermediate FRET contrast, suggesting that they retained some bending rigidity, though less than fully complementary DNA. Remarkably, the remaining mismatches (G-G and G-A) displayed FRET contrast similar to that of fully complementary DNA, indicating that these mismatches were not appreciably more flexible than fully complementary DNA. The FRET data were also broadly consistent with the trends in electrophoretic mobility, confirming that the effects were structural and not photophysical.

The MWLC model predicts that thermodynamically less stable mismatches should kink more easily. Alternate models, in which kinking arises through a structural deformation other than local melting, do not require such a correlation. Flexibility was significantly correlated with the literature values for the thermodynamic penalty of the mismatches (35) (Spearman’s *ρ* = 0.90; *P* = 2.2 × 10^−3^; Figure 3B). This observation strongly supports the MWLC model of kinking.

The relative propensity of the different mismatches to kink also correlated with their biological activity. T4 endonuclease VII, thought to cut at ‘kinkable’ sites, was reported to show low activity on all G-mismatches, intermediate activity on A-A, C-A, C-T and T-T mismatches, and highest activity on C-C mismatches, in direct correspondence with the flexibilities we measured (33). The most flexible mismatch in our assay, C-C, is also the least efficiently repaired by the *E. coli* mismatch repair system (3). Thus the nonlinear mechanical properties of mismatched DNA play an important role in its interaction with DNA-processing enzymes.

### Biochemical probes of kinked DNA

**Figure 4.**
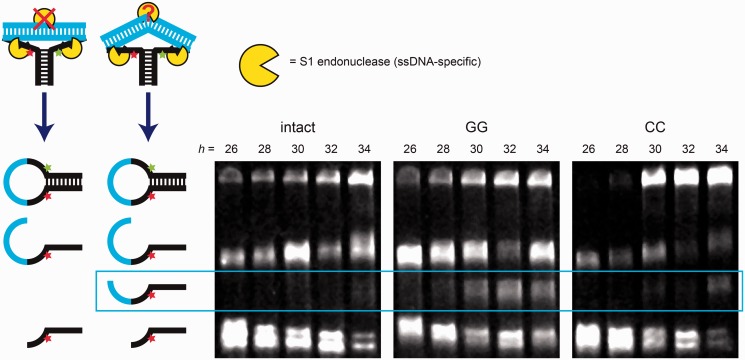
Cleavage of kinked target strands in a molecular vise by S1 endonuclease at high ionic strength. Molecular vises with a 36-nt loop and varying length target strands were treated with the single-stranded DNA-specific S1 nuclease in a high-ionic-strength buffer and analyzed by denaturing PAGE. The target strands were either fully complementary (left image) or contained a single GG (center image) or CC (right image) mismatch. The different complexes reacted at different rates; the cleavage reactions were quenched at 135 min (intact, left), 15 min (GG, center) or 45 min (CC, right). Cartoons on the left depict cleavage products and reactants. The longest band (top) was the undigested hairpin. Of the cleavage products, the longest resulted from cleavage of the loop at its 3′ end; the shortest resulted from cleavage of the loop at its 5′ end; and the middle band, when present, resulted from cleavage within the target strand. This band appeared weakly in the intact target strand of length 34 bp, and prominently in the mismatched target strands of length >30 bp. Cleavage within the target strand indicated kinking-induced disruption of base pairing.

The MWLC model predicts that kinking of DNA involves melting of the base pairs at the site of the kink. To test this prediction, we probed for disruption of base pairing in kinked DNA, examining putative kinks in fully complementary DNA as well as kinks in DNA containing single base-pair mismatches. The single-strand-specific S1 endonuclease is reported to cleave dsDNA in regions where the base-paired double-helical structure has been disrupted (12,36). We added S1 endonuclease to molecular vises with a loop of length 36 and a range of fully complementary and mismatched target lengths, under ionic conditions that favored kinking (‘Materials and Methods’ section). Cleavage products were resolved by denaturing PAGE (Figure 4). S1 readily cleaved molecular vises in the single-stranded regions of the loop flanking the target strand. Under kinking-induced local melting, S1 also cleaved the dsDNA at the location of the kink.

Under high salt conditions, S1 cleaved the perfectly complementary target of length 34 bp, but not shorter targets. These results confirm disruption of base pairing in the strongly kinked state. We infer that for shorter targets, kinking was insufficient to sensitize cleavage to S1 nuclease, either because the melting in the kinked state was incomplete or transient, or because our assay was not sensitive enough to detect a low level of cleavage. Inclusion of a single G-G or C-C base-pairing mismatch sensitized target strands of length 30 bp or higher to S1 cleavage, indicating disruption of base pairing under high curvature of mismatched DNA. Cleavage rates were much greater for the mismatched DNA than for the intact kinked DNA, suggesting a greater extent of structural disruption in the mismatches, consistent with the MWLC model.

## DISCUSSION

The energy to distort a strand of DNA is a functional of its local curvature, *c*(*s*), and torsion, *τ*(*s*), where *s* is the contour coordinate along the molecule
(4)
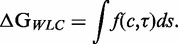



The integrand, *f*(*c*,*τ*), can have a complicated dependence on *c* and *τ*, and in general depends on the temperature, chemical environment and underlying DNA sequence. The WLC model amounts to approximating *f*(*c*,*τ*) by a paraboloid. This approximation must be valid for sufficiently small curvature, and must break down at sufficiently high curvature.

Thermal fluctuations can only generate small deviations from equilibrium curvature; far larger deviations are attained in many actively produced DNA conformations in the cell. Hybridization of short oligonucleotides to a small ring of DNA has previously been used to probe DNA bending at high curvature (37,38), but in these experiments the nonlinear elastic properties of the single-stranded region of the ring complicated interpretation of the results. The key merit of the molecular vise geometry is that it generates high curvatures under well-defined forces, with an unambiguous fluorescence readout of molecular conformation.

**Figure 5.**
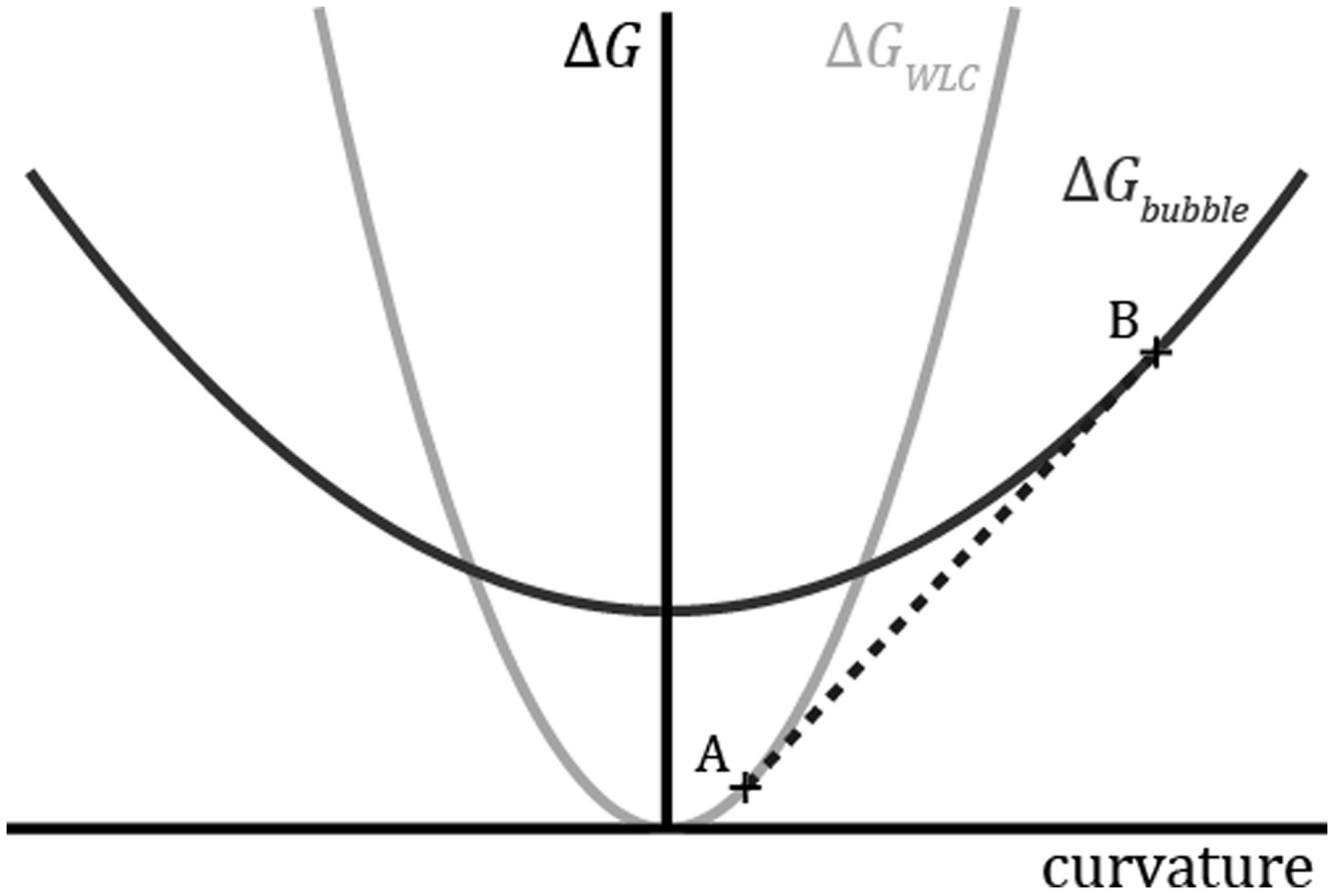
Energy landscape of the MWLC model. The WLC model (gray) approximates the bending energy as a quadratic function of the curvature. In the MWLC model, bubbles of locally melted DNA can form. The bending energy of a bubble (black) is also modeled as a parabola, offset from the WLC curve by the amount of energy required to form the bubble and with shallower slope due to the increased flexibility of the melted region. If the average curvature of the entire strand is constrained to be between the points labeled A and B in the plot, then it is energetically favorable for the strand to kink, with a fraction of the sequence melted and of curvature B, and the remainder intact and of curvature A. The dashed line, which is tangent to both of the curves, corresponds to the bending energy of a kinked strand as a function of its mean curvature.

Under conditions of constant curvature, kinking is a signature of breakdown in the parabolic approximation of *f*(*c*,*τ*). Formation of a kink implies that the mean curvature has reached a value where *f*(*c*,*τ*) is concave. In such a region, the total energy is lower when the molecule separates into regions of high curvature and low curvature, rather than maintaining the mean curvature throughout. Figure 5 illustrates how a concave region of *f*(*c*,*τ*) arises naturally in the MWLC model. At low curvature, the fully base-paired DNA is more stable; at high curvature, the energetic cost of melting is compensated by the decreased energetic cost of bending locally melted DNA.

The existence of a ‘critical curvature’ for kinking has recently been postulated in an effort to reconcile disparate experiments on sharply bent DNA (10). However, the propensity of a molecule to kink is not purely a function of local curvature; rather, it depends on the relative free energies of the kinked and unkinked conformations of the entire molecule, whose elastic components are given by the nonlocal formula, Equation 4. The energy released by a kink can have significant contributions from changes at large distances in bending, base pairing, and single-stranded stretching. The ‘critical curvature’ depends on the context, even when bending is the only contribution to the energy.

A simple example illustrates this point. Consider a molecule of length *L* (much shorter than the persistence length) bent along an arc with constant radius of curvature *R*. Under the WLC approximation, the elastic energy stored in this configuration is
(5)
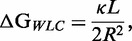

where *κ* is the bending modulus. Introduction of a flexible kink in the middle of the arc completely relaxes the curvature, so the energy of the kinked conformation is only the energy to nucleate the kink, Δ*G_kink_*. Equation 5 shows that the relative values of Δ*G_kink_* and Δ*G_WLC_* depend not just on the radius of curvature, *R*, but also on the contour length of the strand, *L*: longer strands kink at larger radii of curvature.

Introduction of a single fully flexible kink in a DNA minicircle relaxes the molecule to a ‘teardrop’ shape whose energy is numerically calculated to be 28.8% lower than the elastic energy of the circular conformation. (We ignore torsion for simplicity.) Formation of a second kink diametrically opposite the first releases the remaining 71.2% of the elastic energy. Thus, the state with a single kink is unstable to formation of a second kink. In the limit of highly flexible kinks, minicircles with a single kink are predicted not to exist.

Nuclease digestion experiments by Du and Vologodskii showed a disruption of the double-helix in minicircles with contour length of 64–65 bp (12). In the circular conformation, the elastic energy is (Equation 5) Δ*G_WLC_* ∼43 *k_B_T*. 28.8% of this elastic energy can be released by forming a single kink. A recent analysis by Vologodskii and Frank-Kamenetskii thereby concluded a kink energy of ∼12 *k_B_T* (10). However, if one considers the formation of two kinks simultaneously, then 50% of the elastic energy is available for each kink, implying a kink energy of ∼21 *k_B_T*, in remarkably close agreement with our results.

Torsional strain significantly affects energetics of minicircles, but not DNA vises, and this difference could account for the remaining difference between the kink energies reported by the two techniques. Residual curvature in doubly kinked minicircles, for example due to imperfect kink flexibility, would also lower the estimate of the kinking energy for minicircles. Finally, the kink energy may depend on buffer composition, a factor that differed between our experiments and those of Du and Vologodskii (12).

Our results provide insight into the controversies and apparent discrepancies among previous measurements of DNA bending. All reported bulk-scale solution cyclization experiments have been consistent with the WLC model (2), with two exceptions. The validity of the first (39) has been questioned (23) due to its use of possibly saturating ligase concentrations. The results of the second (30) were consistent with a MWLC model.

The recent single-molecule cyclization results of Vafabakhsh and Ha (13) suggested that short unconstrained DNA strands are significantly more flexible than the WLC prediction. These experiments were performed in buffers containing 10 mM magnesium or >500 mM sodium and probed rare thermal excursions to regions of high curvature. Our results suggest that this observed increase in DNA flexibility at high curvature may be due to the high ionic strength, which may facilitate local melting. Two other studies observed kinking of short pieces of DNA in minicircles in buffers containing magnesium or other divalent cations (12,38). A computational model of counterion condensation predicted that kinked structures would be better stabilized in high ionic strength solution, particularly when the cation was Mg^2+^ (40).

## CONCLUSION

Molecular vises, in combination with our simple statistical mechanical model, are a powerful platform for studying DNA bending mechanics. While we have focused on intact DNA and single-nucleotide mismatches, one could use the same approach to study the effects of many other perturbations. For instance, mechanical changes may arise from variations in the target sequence [e.g. inclusion of A-tracts (41)], epigenetic modifications [e.g. methylation of cytosine (42) or adenine (43)], damage [e.g. thymine dimers (44) or 8-oxoguanine], DNA-modifying drugs [e.g. cisplatin (45) or doxorubicin (46)] or hybridization with RNA.

Molecular vises might also be used to investigate the role of DNA mechanics in protein–DNA interactions. Two classes of effects may be investigated. First, pre-bending of DNA may affect its interactions with proteins, particularly when the bound state or a reaction intermediate involves bending the DNA. For instance, recognition of mismatches by the MutS protein is thought to depend on the equilibrium between bent and unbent states of the protein–DNA complex (31,47). Using a molecular vise to bias mismatched DNA toward the bent state might modulate binding and recognition.

Second, molecular vises could report on the conformational changes induced in DNA by protein binding. Numerous proteins, such as transcription factors and architectural modulators, bend the DNA they bind (2,48,49) and therefore might directly alter the FRET efficiency of a molecular vise. Additionally, the compressive force applied by the molecular vise ‘pre-stresses’ the binding site so that even a small increase in flexibility on protein binding might be amplified into a large change in FRET.

A particularly useful feature of molecular vises and other FRET-based probes of protein binding (50,51) is that the protein itself does not need to be labeled. For sequence-specific DNA-binding proteins, one could design a molecular vise containing the binding site in the target strand. Protein binding would lead to a change in FRET. This concept is similar to the idea underlying molecular beacons for detecting unlabeled short oligonucleotides. Finally, we note that the molecular vise structure and the FRET-based readout are amenable to single-molecule studies that may probe transient DNA–protein interactions or sparsely populated states.

## SUPPLEMENTARY DATA

Supplementary Data are available at NAR Online, including [52–55].

## FUNDING

NSF [CHE-0910824]; Dreyfus Teacher Scholar Award; Sloan Foundation Fellowship. Funding for open access charge: NSF.

*Conflict of interest statement*. None declared.

## Supplementary Material

Supplementary Data
