# Supplementary material for: Euler buckling and nonlinear kinking of double-stranded DNA
Source: Nucleic Acids Res. 2013 Aug 16;41(21):9881–90. doi: 10.1093/nar/gkt739 (PMC3834817; doi:10.1093/nar/gkt739)
Supplement: Supplementary Data [file supp_41_21_9881__index.html]

Euler buckling and nonlinear kinking of double-stranded DNA — Euler buckling and nonlinear kinking of double-stranded DNA — Supplementary Data 

# Euler buckling and nonlinear kinking of double-stranded DNA

## Supplementary Data

files

**Files in this Data Supplement:**

- Supplementary Data - pdf file
