# Supplementary material for: Euler buckling and nonlinear kinking of double-stranded DNA
Source: Nucleic Acids Res. 2013 Aug 16;41(21):9881–90. doi: 10.1093/nar/gkt739 (PMC3834817; doi:10.1093/nar/gkt739)
Supplement: Supplementary Data [file supp_gkt739_nar-01556-f-2013-File007.pdf]

Supporting Information
Euler buckling and nonlinear kinking of double-stranded DNA

Alexander P. Fields[a], Elisabeth A. Meyer[b], Adam E. Cohen[b,c*]
[a]*Biophysics Program*, [b]*Department of Chemistry and Chemical Biology*, [c]*Department of Physics, 12 Oxford St, Harvard University, Cambridge, MA 02138*
[*]*To whom correspondence should be addressed (cohen@chemistry.harvard.edu, tel 617-496-9466)*

Supporting Materials and Methods

FRET Data Analysis. All image analysis and computations were performed using MATLAB R2012a (The MathWorks, Inc.). We wrote custom software to extract FRET efficiencies from gel images with high precision. Bands were selected manually from gel images. A 5 pixel × 5 pixel median filter was applied to each image to remove speckle. For each band, we plotted pixel-by-pixel A_{GR} and A_{GG} as a function of A_{RR}. Using the MATLAB "robustfit" function with default parameters we determined the slopes of these lines: I_G for green emission, and I_R for red emission. This regression procedure suppressed the influence of local background compared to simple integration of the intensities of each channel, resulting in an improved signal-to-noise ratio. FRET efficiencies were calculated using the slopes, corrected for experimentally measured dye crosstalk and relative dye brightness according to

$$E_{FRET} = \frac{I_R - X_{AR} - I_G X_{DR}}{I_R - X_{AR} - I_G X_{DR} + \rho I_G}, \quad \textbf{[S1]}$$

where X_{AR} is the ratio of the emission of the acceptor (Alexa 647) in the red detection channel under green versus red excitation, X_{DR} is the ratio of the emission of the donor (Cy3B) into the red versus green detection channel, and ρ is the ratio of the emission of the acceptor dye into the red channel to that of the donor dye into the green channel. Emission of the acceptor dye in the green detection channel and excitation of the donor dye by the red laser were both determined experimentally to be negligible. Note that these parameters depend not only on the properties of the dyes themselves, but also on the instrument used to perform the spectroscopy. X_{AR} and X_{DR} were measured using singly labeled molecules, and ρ was inferred from the hairpin data themselves via a Deming regression of I_R against I_G, the slope of which was taken to be $X_{DR} - \rho$. FRET efficiencies were further corrected for incomplete acceptor-dye labeling by dividing by the value measured in the absence of a complementary strand, which would be expected to display 100% FRET efficiency. This correction was applied for each hairpin type separately to account for variations in labeling or purification efficiency.

Statistical Mechanical Model. The partition function includes degrees of freedom corresponding to the extent of base-pairing in the stem and target strand, as well as the end-to-end distance (i.e. amount of bending) of the target strand:

$$Z = \sum_{n_{stem}=0}^{20} \sum_{n_A=\frac{n_L}{2}-\left\lfloor\frac{n_h}{2}\right\rfloor}^{n_L/2} \sum_{n_D=\frac{n_L}{2}-\left\lceil\frac{n_h}{2}\right\rceil}^{n_L/2} \int_{r_{min}}^{r_{max}} \mathrm{d}r\, z[r, n_{stem}, n_A, n_D, n_L] \quad \textbf{[S2]}$$

$$z[r, n_{stem}, n_A, n_D, n_L] = \exp\left[-\frac{1}{k_B T}\left(G_{bp}^{stem} + G_{bp}^{loop} + G_{ss} + G_{rod}\right)\right]$$

where n_{stem}, n_A, and n_D are the number of *unpaired* bases in the stem, the side of the loop closer to the acceptor dye (5′ side), and the side of the loop closer to the donor dye (3′ side), respectively; r is the end-to-end distance of the duplex target strand; r_{min} = 1.8 nm is the displacement between the two base-paired strands at the end of a DNA double helix; n_L is the number of nucleotides in the loop; and n_h is the number of nucleotides in the complement strand. We truncate the sum at n_{stem} = 20 because successive terms contribute negligibly. The base-pairing free energies G_{bp} are each calculated using sums of nearest-neighbors dinucleotide energies from laser tweezers unzipping experiments (19) and the relevant sequences (with loss of hybridization given by n_{stem} or n_A and n_D).

We model the single-stranded region as a freely jointed chain (52); ignoring constant terms, we have

$$G_{ss} \approx \frac{L_{ss}}{b}\left(f_{ss}\mathcal{L}^{-1}(f_{ss}) - \ln\left(\frac{\sinh \mathcal{L}^{-1}(f_{ss})}{\mathcal{L}^{-1}(f_{ss})}\right)\right) \quad \textbf{[S3]}$$

$$L_{ss} = l_{ss}(2n_{stem} + n_A + n_D)$$

$$f_{ss} \equiv \frac{r_{ss}}{L_{ss}} = \frac{r - r_{min}}{L_{ss}}$$

$$\mathcal{L}(y) \equiv \coth y - \frac{1}{y}$$

where L_{ss} is the contour length of the single-stranded portion of the loop, including unzipped bases from the stem. The parameters for this model are the Kuhn length (b) and the per-nucleotide contour length (l_{ss}) of single-stranded DNA. Salt-dependent values for both parameters are available from the same reference as the base-pairing energies (19). To simplify integration, we approximate the inverse Langevin function as

$$\mathcal{L}^{-1}(f_{ss}) \approx \frac{f_{ss}(f_{ss}^2 - 3f_{ss} + 3)}{1 - f_{ss}}. \quad \textbf{[S4]}$$

The equilibrium conformation of a rigid rod buckled by compressive force is given by a set of curves known as elastica; their associated bending energy is (17, 53)

$$G_{rod} = \frac{8\kappa K^2(m)}{L_{ds}}\left(\frac{f_{ds} - 1}{2} + m\right) \quad \textbf{[S5]}$$

$$L_{ds} = l_{ds}(n_L - n_A - n_D)$$

where $K(m)$ is the complete elliptic integral of the first kind, L_{ds} is the contour length of the double-stranded portion of the loop, l_{ds} is the per-nucleotide contour length of double-stranded DNA, and the parameter $m \geq 0$ is the solution to

$$f_{ds} \equiv \frac{r}{L_{ds}} = 2\frac{E(m)}{K(m)} - 1 \quad \textbf{[S6]}$$

where $E(m)$ is the complete elliptic integral of the second kind. The value of m specifies the conformation of the rod. When $m = 0$, the rod is fully extended ($f_{ds} = 1$); as m increases, the rod becomes increasingly bent. We simplify the integration by approximating

$$m \approx (1 - f_{ds})(M + (4M^2 - 3M)f_{ds} + (1 + 2M - 4M^2)f_{ds}^2) \quad \textbf{[S7]}$$

where $M = 0.8261\ldots$ is the value of m at which the rod is maximally bent, such that its ends are touching (i.e., when $f_{ds} = 0$). We neglected fluctuations in degrees of freedom beyond the principal flexural mode. The energy and equilibrium conformation of a buckled rod (of constant contour length) is plotted as a function of end-to-end distance in Fig. S1.

Observables are predicted using the appropriate partition sums and integrals. The ensemble average FRET efficiency is predicted by

$$E_{FRET} = \frac{1}{Z}\sum_{n_{stem}=0}^{20}\sum_{n_A=\frac{n_L}{2}-\left\lceil\frac{n_h}{2}\right\rceil}^{n_L/2}\sum_{n_D=\frac{n_L}{2}-\left\lceil\frac{n_h}{2}\right\rceil}^{n_L/2}\int_{r_{min}}^{r_{max}} \mathrm{dr}\, \frac{z[r, n_{stem}, n_A, n_D, n_L]}{1+\left(\frac{r_{min}+f_{dye}(r-r_{min})}{R_0}\right)^6} \quad \textbf{[S8]}$$

where R_0 is the Förster radius of Cy3B and Alexa 647, and

$$f_{dye} \equiv \frac{1+2n_{stem}}{2n_{stem}+n_A+n_D} \quad \textbf{[S9]}$$

represents the ratio of the distance between the dyes to the distance between the ends of the target strand. Eq. **S9** models the dyes as separated by a fixed fractional distance of the total single-stranded DNA extension, neglecting any fluctuations other than the end-to-end fluctuations of the entire chain. We used a range of values for R_0 (Table S1) within a previously estimated range for Cy3B and Cy5 (54) to account for the uncertainty both in its value and in the other parameters and assumptions in the model.

Partition integrals were calculated numerically using the MATLAB "quad" function with default settings, on an Intel Core i7 notebook computer running 64-bit Windows 7. Partition terms were evaluated for each hairpin for each degree of base pairing in the stem and loop, and appropriate terms were summed for each target strand length (Eq. **S2**). A range of FRET values were calculated using the range of R_0 values in Table S1, and the results for $R_0 = 6.2$ were fit to experimental data for each hairpin individually using a one-parameter least-squares regression, in which the offset was fixed such that the value $E_{FRET} = 1$ remained unchanged. This scaling was then applied to the range of FRET results and plotted as the shaded areas in Fig. 1.

The model allowed us to validate certain aspects of the vise design. We tested computationally whether the ends of the target strand remained hybridized or whether they unzipped under compressive load. When the target strand contained A-T base pairs near its ends, the model predicted partial fraying of the ends. However, when the target contained all G-C base pairs outside the central 18 (as was the case in our experiments), then fraying was eliminated.

This statistical mechanical model focuses on the key mechanical aspects of the molecular vise but ignores numerous other details, such as fluctuations in the buckled rod or single-stranded DNA beyond the principal degree of freedom; torsional effects in the target strand; the length and fluctuations of the dye linkers; the relative orientation of the dyes; the details of base-pairing beyond the nearest-neighbors model; sequence-specificity in dsDNA or ssDNA bending

moduli; and electrostatic interactions between different parts of the construct and each other or surrounding ions. An atomistic model might better account for these effects, at the expense of significant mathematical and computational complexity.

Sequences. All are written 5′ to 3′

Loop sequences (30, 36, 40, 46, and 50 nt):

```
TCGCCCACCGATAAGCTTGGTCATGCCCGT
TGCCCGCCCACCGATAAGCTTGGTCATGCCCGCCGT
TCCGCCCGCCCACCGATAAGCTTGGTCATGCCCGCCGCCT
TCCGCCGCCCGCCCACCGATAAGCTTGGTCATGCCCGCCGCCGCCT
TGCCCGCCGCCCGCCCACCGATAAGCTTGGTCATGCCCGCCGCCGCCCGT
```

The outermost thymine nucleotides were amine-modified and linked to Alexa647 (5′ side, red) or Cy3B (3′ side, green). Complementary strands did not include base-pairing partners for the dye-conjugated nucleotides. Site specific mismatches were placed at the center of the target strand, opposite the positions marked in blue.

Stem sequence:

```
GCCCGGCGGCTTATAAAATTTATTAATTATATATTTTATTTAATATAAT-Loop
```

Complementary sequences (20–48 nt):

CATGACCAAGCTTATCGGTG

GCATGACCAAGCTTATCGGTG

GCATGACCAAGCTTATCGGTGG

GGCATGACCAAGCTTATCGGTGG

...

CGGGCGGCGGCGGGCATGACCAAGCTTATCGGTGGGCGGGCGGCGGGC

Mismatched target strands contained single base substitutions at one of the nucleotides marked in blue (the T was replaced with A for the A-A mismatches, the A was replaced with T for the T-T mismatches, and the central nucleotides were replaced as appropriate to generate the other six mismatches).

Supporting references

52. Wang,M.C. and Guth,E. (1952) Statistical theory of networks of non-gaussian flexible chains. *J. Chem. Phys.*, **20**, 1144-1157.

53. Emanuel,M., Mohrbach,H., Sayar,M., Schiessel,H. and Kulic,I.M. (2007) Buckling of stiff polymers: Influence of thermal fluctuations. *Phys. Rev. E. Stat. Nonlin Soft Matter Phys.*, **76**, 061907.

54. Uphoff,S., Holden,S.J., Le Reste,L., Periz,J., Van De Linde,S., Heilemann,M. and Kapanidis,A.N. (2010) Monitoring multiple distances within a single molecule using switchable FRET. *Nat. Methods*, **7**, 831-836.

55. Narayana,N. and Weiss,M.A. (2009) Crystallographic analysis of a sex-specific enhancer element: Sequence-dependent DNA structure, hydration, and dynamics. *J. Mol. Biol.*, **385**, 469-490.

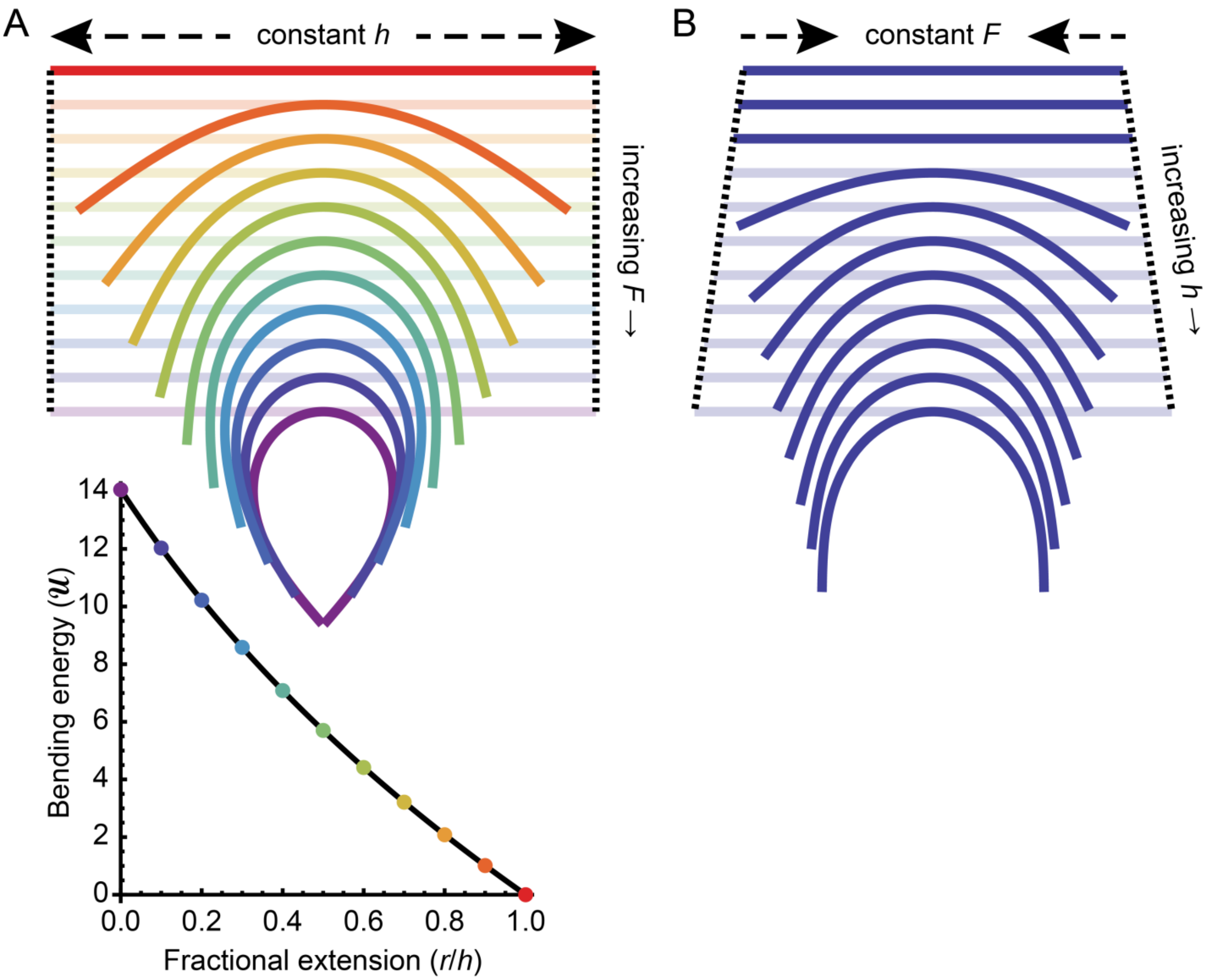

Fig. S1. Classical mechanics of Euler buckling of rigid rods under compressive force ("elastica"). (*A*) Top: rods of constant length under increasing compressive force. Bottom, bending energy as a function of fractional end-to-end extension. Colored points correspond to the same-colored configurations above. Dimensionless bending energy is defined $\mathcal{U} \equiv U\, h/\kappa$, where h is the contour length and κ is the bending modulus. In these units the energy to bend a rod into a perfect circle is $\mathcal{U} = 2\pi^2$. (*B*) Conformations of rods of increasing length under constant compressive force. These shapes correspond to DNA of length 40–50 bp experiencing a compressive force of 9 pN, in the absence of thermal effects. The buckling transition occurs between 42 and 43 bp.

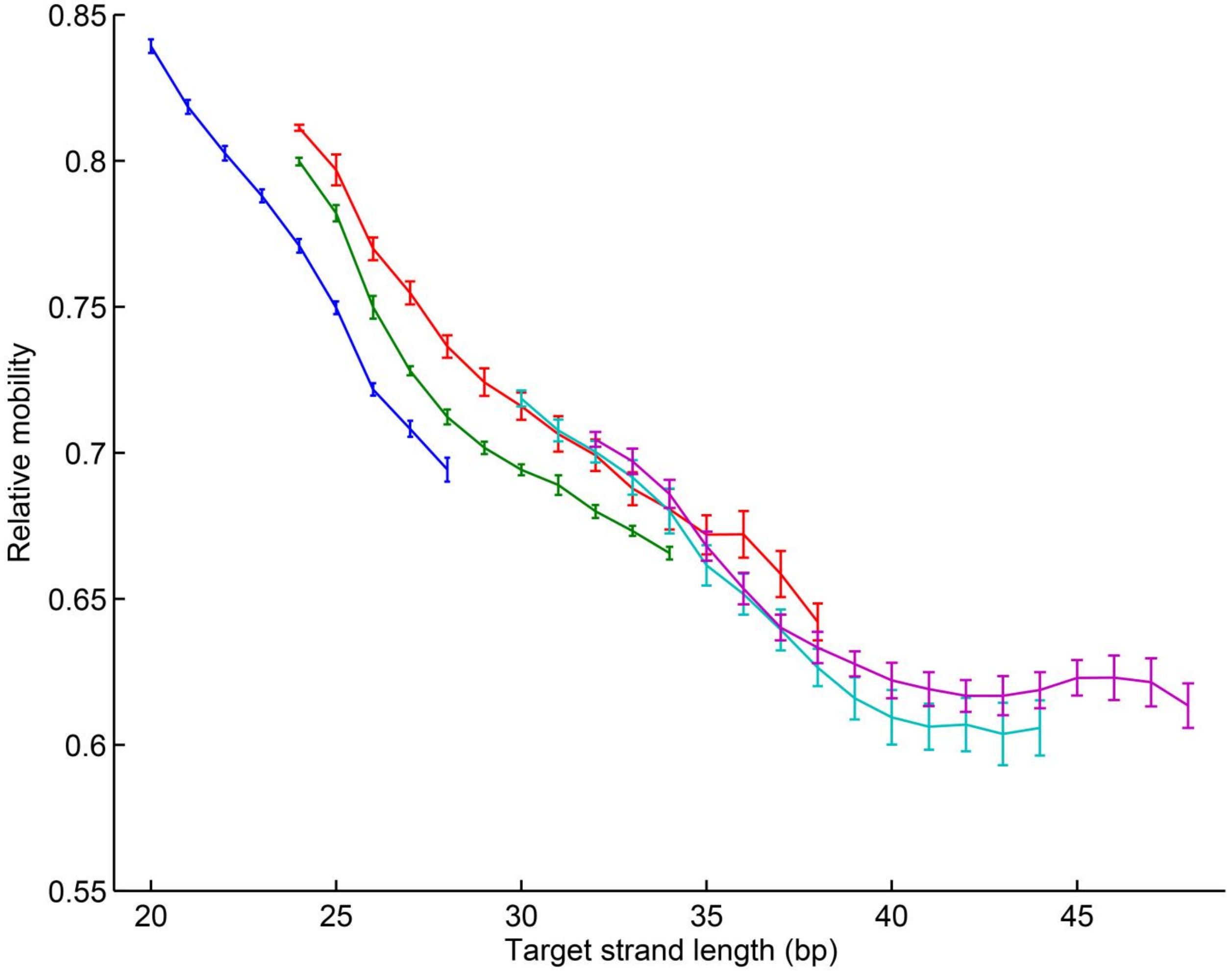

Fig. S2. Relative electrophoretic mobility of five loop sizes of molecular vises as a function of target strand length (colors as in Fig. 1*C*). Relative mobility was defined as the distance traveled by the complex divided by the distance traveled by the molecular vise in the absence of a complementary strand (i.e., the hairpin only). Below the buckling transition ($L = 40$), the relative mobility decreased with increasing target strand length; beyond the buckling transition, increasing target strand length did not significantly affect the relative mobility, suggesting that the complex adopted an increasingly compact structure.

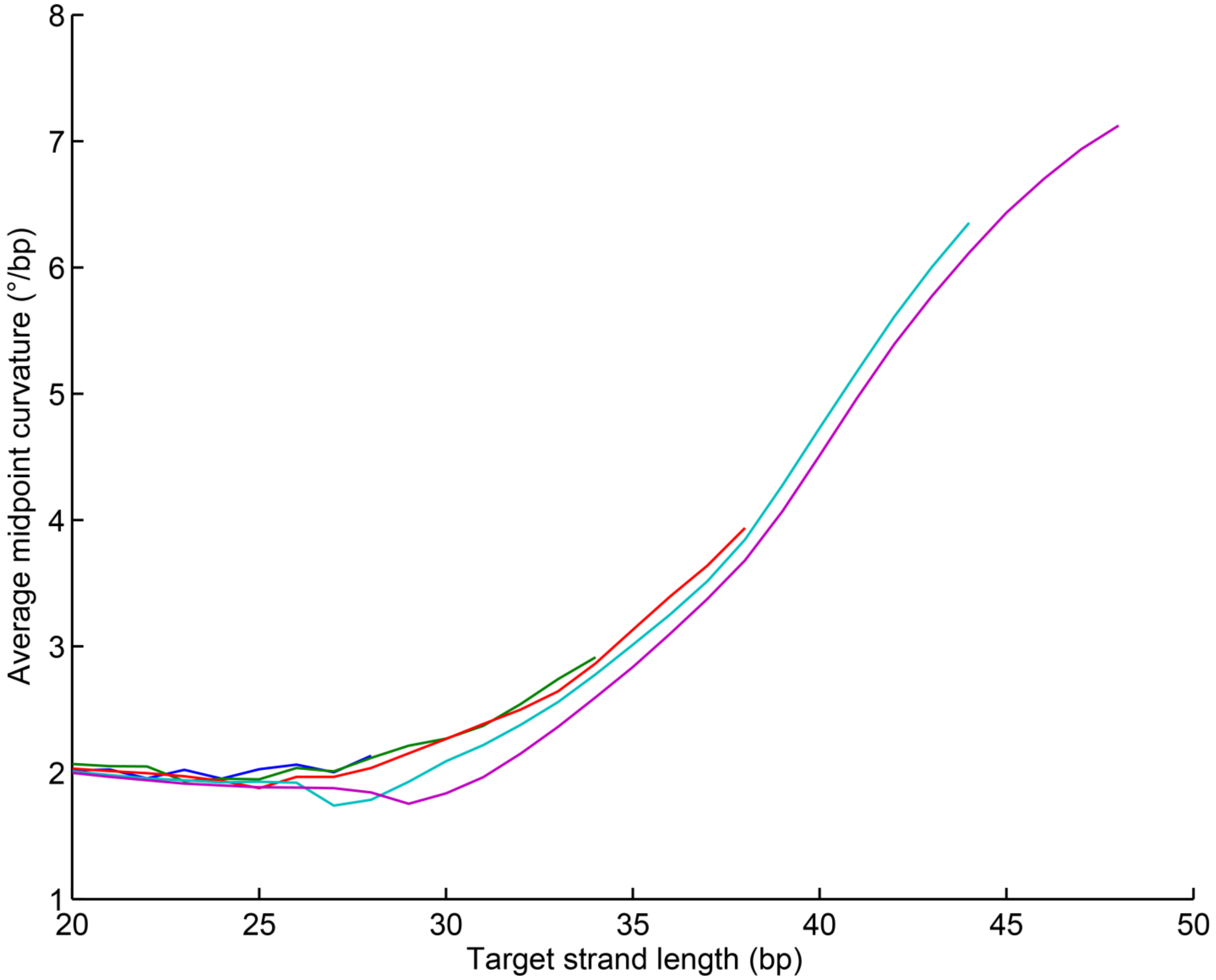

Fig. S3. Predicted ensemble average curvature at the apex of molecular vises (the midpoint of the target strand) of five loop sizes (colors as in Fig. 1*C*). In the absence of thermal effects, the curvature would be zero when the target strand is shorter than the buckling length (40 bp), but would rise sharply at longer lengths (Fig. S1*B*). According to our statistical mechanical model, thermal fluctuations smooth out the buckling transition so that the angle increases gradually. We also predict that thermal fluctuations produce a baseline average curvature of 2°/bp for short target strands. The maximum average curvature that our model predicts for any of our constructs is 7°/bp.

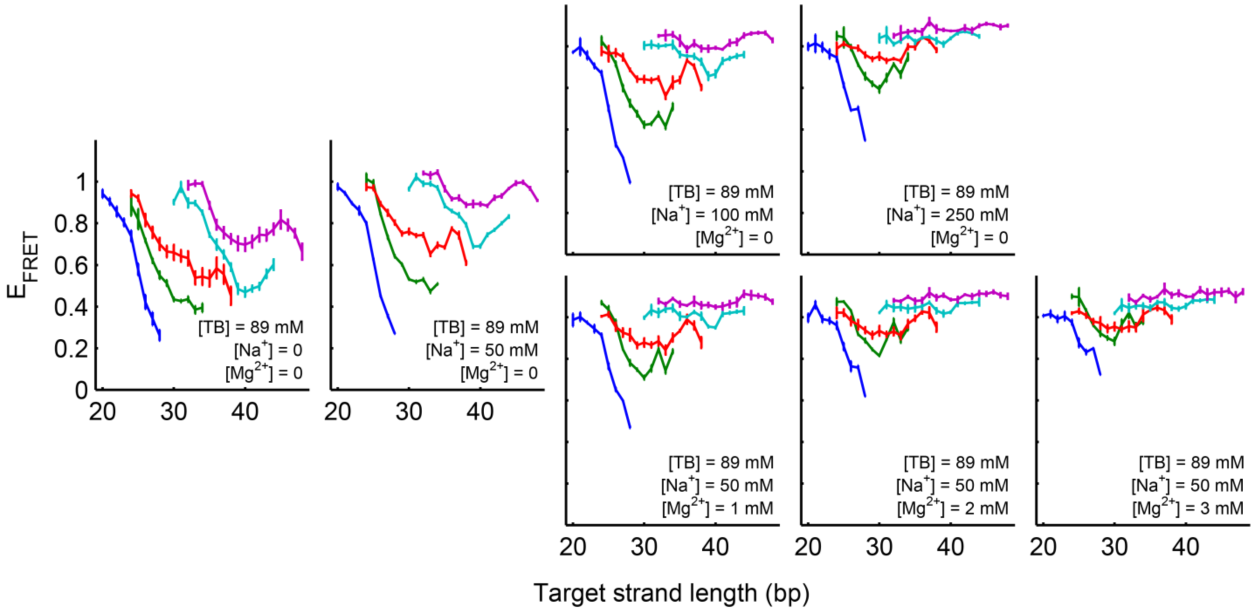

Fig. S4. Effect of ionic strength on duplex DNA bending in molecular vises: full results. Shown here are the FRET efficiencies of the five molecular vises (colors as in Fig. 1*C*) at all target strand lengths at all tested salt mixtures, extending the plots shown in Fig. 2. Inscriptions list all buffer components other than deionized water; "TB" means equimolar tris/borate. The coexistence of the two bending transitions, buckling (40 bp) and kinking (30–33 bp), was most evident at [Na^+] = 100 mM (center, top) or at [Na^+] = 50 mM, [Mg^{2+}] = 1 mM (center, bottom). Buckling was dominant at lower ionic strength, but was replaced by kinking at higher ionic strength.

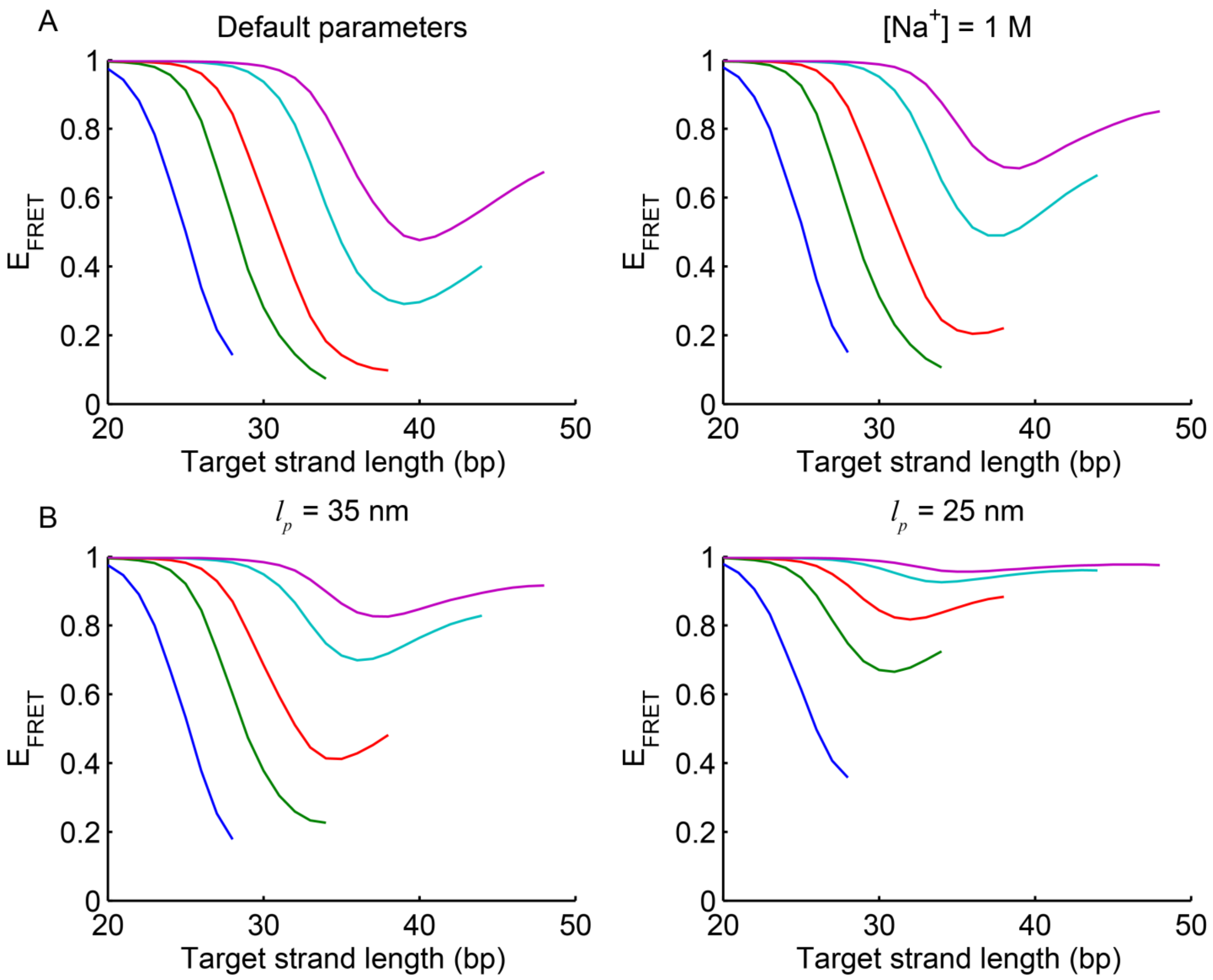

Fig. S5. Effects of model parameters on predicted FRET curves. (*A*) Increasing the base-pairing energies and the flexibility of single-stranded DNA to the values at 1 M monovalent salt (19) only slightly shifted the expected buckling length and did not explain the appearance of a bending transition at 30 bp at high ionic strength (Fig. 2 and Fig. S4). (*B*) To achieve a buckling transition at 30 bp, the double-stranded DNA persistence length had to decrease from 46.5 nm to 25 nm, a physically implausible value. The model predicted the shift from buckling at 40 bp to buckling at 30 bp to be gradual: intermediate conditions yielded intermediate transition lengths. Experimentally, we observed the coexistence of two distinct bending transitions without intermediate buckling lengths (Fig. S4), in contradiction to the predictions of the WLC model. Colors in all plots are as in Fig. 1*C*.

Table S1. Parameter values and sources used in the statistical mechanical model of DNA buckling in molecular vises

**Name**	**Symbol**	**Value**	**Source**
dsDNA persistence length	l_p	46.5 nm	(2, 9)
dsDNA rise per base	l_{ds}	0.34 nm	(6, 9)
dsDNA width	r_{min}	1.8 nm	From PDB structure 3BSE (55)
dsDNA base-pairing energies	G_{bp}	various (see ref)	(19) (values for 250 mM salt)
ssDNA Kuhn length	b	1.25 nm	(19)
ssDNA rise per base	l_{ss}	0.59 nm	(19)
Cy3B/Alexa647 Förster radius	R_0	5.7–6.7 nm	(54)

These parameters were used to fit the data in Fig. 1*C*. r_{min} and R_0 were assumed to be independent of salt concentration; for the other parameters, the listed references include salt dependences used in Fig. S5*A*.
